# Supplementary material for: Synthesis, Characterization, Bioavailability and Antimicrobial Studies of Cefuroxime-Based Organic Salts and Ionic Liquids
Source: Pharmaceutics. 2024 Oct 2;16(10):1291. doi: 10.3390/pharmaceutics16101291 (PMC11510342; doi:10.3390/pharmaceutics16101291)
Supplement: Supplementary file 1 [file pharmaceutics-16-01291-s001.zip › pharmaceutics-3124703-supplementary.pdf]

pharmaceutics

# Supplementary Materials

## Synthesis, Characterization, Bioavailability and Antimicrobial Studies of Cefuroxime-Based Organic Salts and Ionic Liquids

**Francisco Faísca[1], Željko Petrovski[1], Inês Grilo[2], Sofia A. C. Lima[3], Miguel M. Santos[1] and Luis C. Branco[1],***

[1]LAQV-REQUIMTE, Department of Chemistry, NOVA School of Science and Technology, NOVA University of Lisbon, 2819-516 Caparica, Portugal.
[2]UCIBIO – Applied Molecular Biosciences Unit, Department of Life Sciences, NOVA School of Science and Technology, NOVA University Lisbon, 2819-516 Caparica, Portugal
[3]LAQV, REQUIMTE, ICBAS - School of Medicine and Biomedical Sciences, University of Porto, Porto, Portugal
*Correspondence: l.branco@fct.unl.pt

## General remarks

Commercially available reagents from Sigma-Aldrich, Alfa Aesar and TCI were purchased from Laborspirit and used as received. Honeywell and standard solvents were also purchased from Laborspirirt and used without further purification.

The basic anion-exchange resin Amberlite 26-OH (ion-exchange capacity 0.8 eq.mL-1) was purchased from Supelco. $^{1}H$ and $^{13}C$ NMR (in APT mode) spectra in $CD_3OD$ (from Euriso-Top) were recorded on Bruker AMX400 and AMX500 spectrometers at 25 °C. To perform NMR, 5 mm borosilicate tubes were used, and the sample concentration was, approximately, 20 mg/mL for $^{1}H$ NMR and 40 mg/mL for $^{13}C$ NMR. Chemical shifts are reported downfield in parts per million (ppm). FTIR spectra were measured on a Perkin Elmer 683 in ATR mode. The elemental analysis experiments were performed in a CHNS Series Thermo Finnigan-CE Instruments Flash EA 1112 under standard conditions (T combustion reactor 900 °C, T GC column furnace 65 °C, multiseparation SS GC column, He2 flow 130 mL/min, $O_2$ flow 250 mL/min) at the Analysis Laboratory LAQV REQUIMTE – Chemistry Department, FCT NOVA, Portugal.

### Methods

All CFX-OSILs were prepared according to the following general procedure.

*General procedure*

One molar equivalent of each cation halide salt (0.5 equivalents in the case of $[PyC_{10}Py]Br_2$) was dissolved in 1-2 mL of methanol and left to stir with 1 mL of anionic resin Amberlyst A-26 hydroxide form (A-26 (OH)). After 1 hour, the solution was filtered and the resin was washed thoroughly thrice with methanol. Meanwhile, Cefuroxime (51 mg, 118 μmol) was dispersed in 2 mL of distilled water, to which 75 mg of ammonium bicarbonate was added under stirring. After complete dissolution, the cations' solutions were added dropwise onto the cefuroxime buffered solution while stirring at 0 °C. The resulting solution was stirred for 15 minutes before it was evaporated in a rotary evaporator at 40 °C and subsequently dried under high vacuum for 24 hours.

**1-Hexadecylpyridin-1-ium (6*S*,7*S*)-3-((carbamoyloxy)methyl)-7-((*Z*)-2-(furan-2-yl)-2-(methoxyimino) acetamido)-8-oxo-5-thia-1-azabicyclo[4.2.0]oct-2-ene-2-carboxylate, [$C_{16}$Py][CFX]**

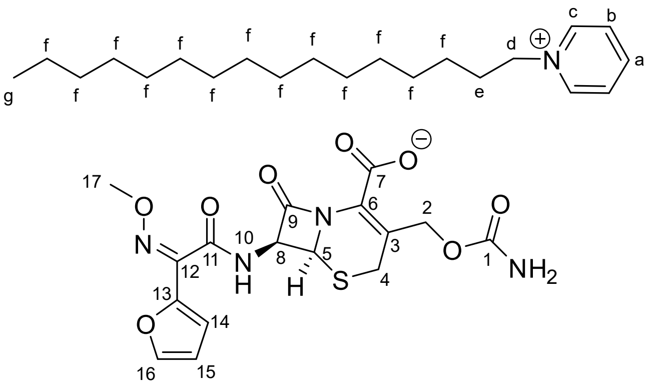

1-Hexadecylpyridin-1-ium hydroxide was prepared by stirring a solution of 1-hexadecylpyridin-1-ium bromide (40.1 mg) with 1 mL of Amberlyst A-26(OH). Upon addition to Cefuroxime, the resulting solution was stirred for 15 minutes before evaporation and subsequent drying to provide the desired product as a dark red solid in quantitative yield (86 mg). IV (KBr) ν 2922, 2852, 1770, 1698, 1600, 1486, 1393, 1319, 1158, 1041, 1009, 885, 745, 684 $cm^{-1}$. $^{1}$H NMR (400 MHz, $CD_3OD$) δ 9.00 (d, J = 5.6 Hz, 2H, C), 8.59 (t, J = 7.8 Hz, 1H, A), 8.12 (t, J = 6.5 Hz, 2H, B), 7.64 (d, J = 14.2 Hz, 1H, 16), 6.77 (d, J = 3.5 Hz, 1H, 14), 6.55 (br s, 1H, 15), 5.78 (d, J = 4.8 Hz, 1H, 8), 5.09 (d, J = 4.7 Hz, 1H, 5), 4.95 (d, J = 12.5 Hz, 1H, 2a), 4.79 (d, J = 12.5 Hz, 1H, 2b), 4.64 (t, J = 7.2 Hz, 2H, d), 3.95 (s, 3H, 17), 3.61 (d, J = 17.8 Hz, 1H, 4a), 3.35 (d, J = 17.8 Hz, 1H, 4b), 2.02 (t, J = 7.2 Hz, 2H, e), 1.44 – 1.15 (m, 26H, f), 0.90 (t, J = 6.2 Hz, 3H, g). $^{13}$C NMR (101 MHz, $CD_3OD$) δ 168.9, 164.4, 164.3, 159.7, 147.1, 146.8, 146.2, 146.2, 145.9, 133.7, 129.5, 116.9, 114.2, 112.8, 65.4, 63.1, 63.1, 60.1, 58.8, 33.1, 32.5, 30.8, 30.7, 30.7, 30.6, 30.5, 30.4, 30.1, 27.2, 26.7, 25.0, 24.13, 23.7, 14.4. Analysis calcd. for $C_{37}H_{53}N_5O_8S(H_2O)_{3.5}$: C, 56.19; H, 7.; N, 8,85; found: C, 56.20; H, 7.95; N, 8.85.

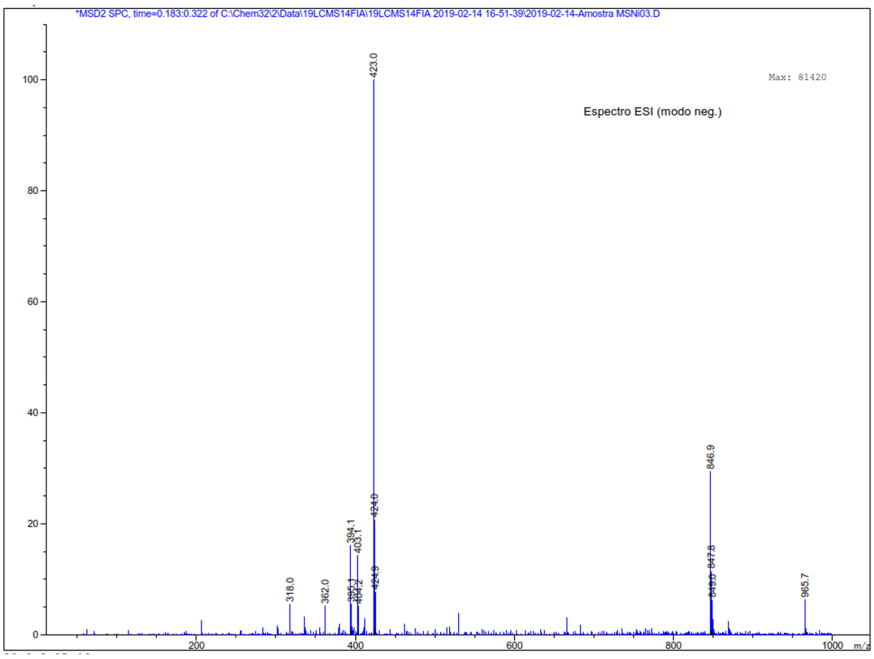


Figure S1. ESI-MS of [$C_{16}$Py][CFX] in negative mode (FIA injection).

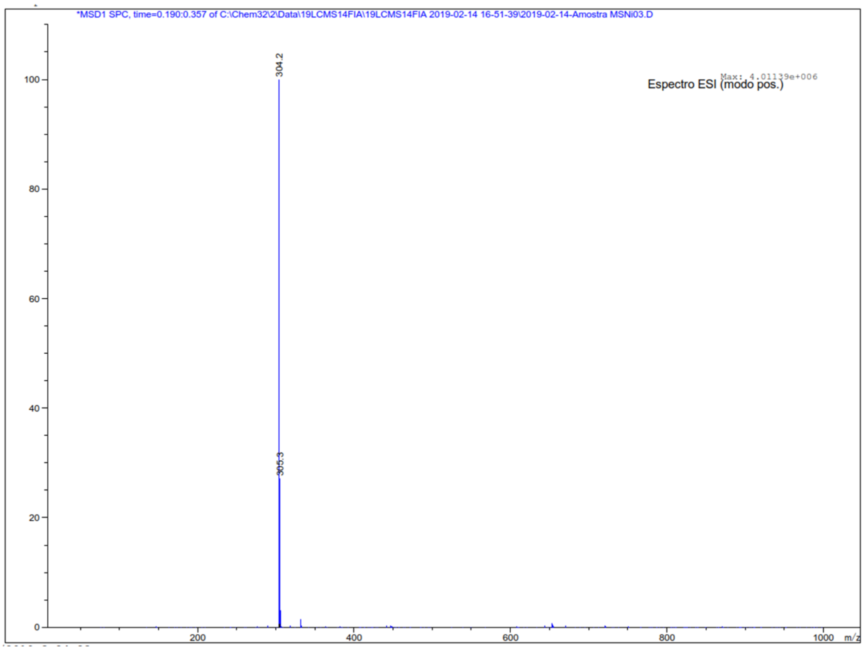


Figure S2. ESI-MS of [$C_{16}$Py][CFX] in positive mode (FIA injection).

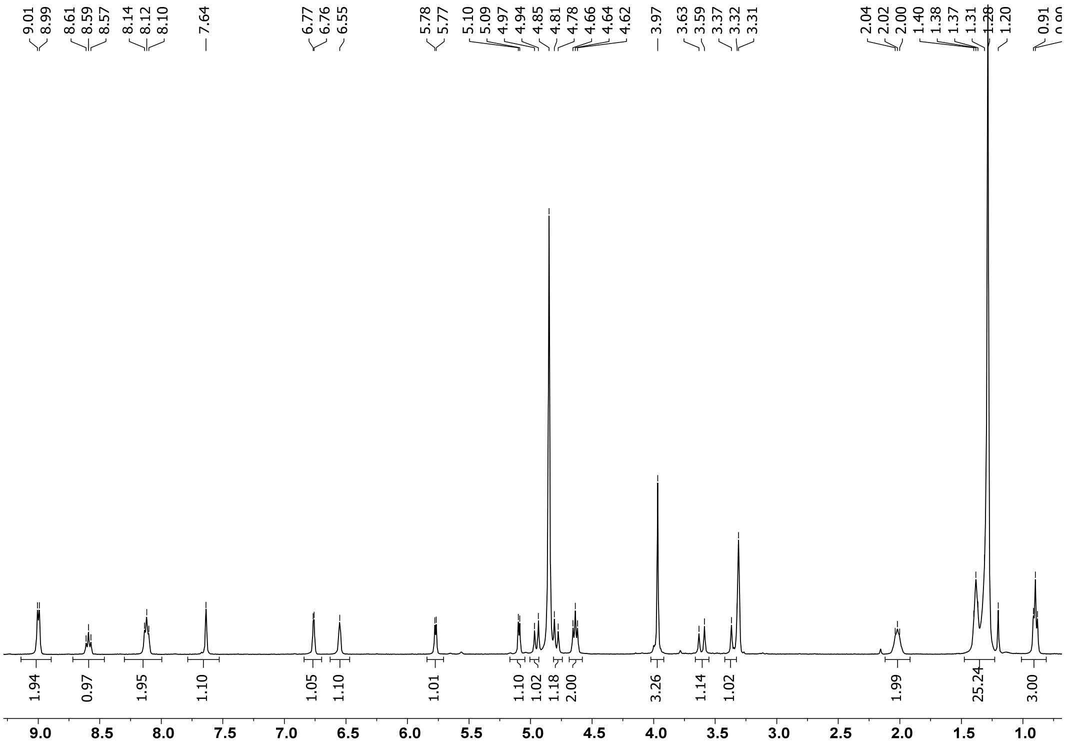


Figure S3. $^{1}$H NMR spectrum of [$C_{16}$Py][CFX] in $CD_3OD$.

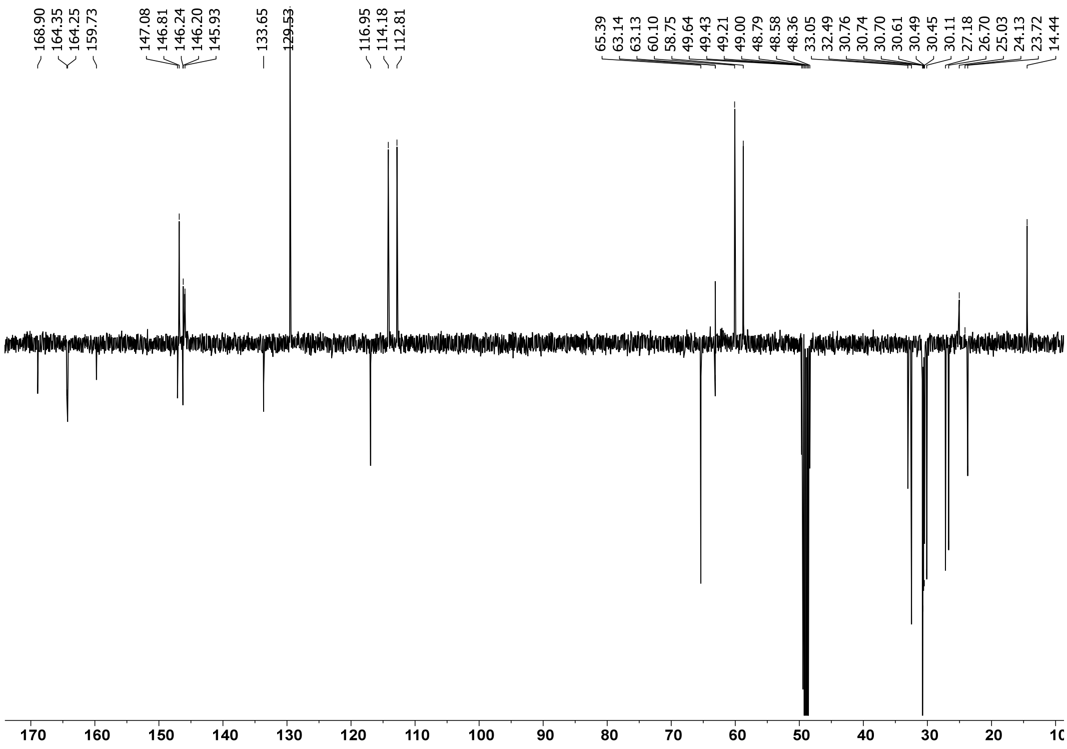


Figure S4. $^{13}C$ NMR-APT spectrum of [$C_{16}$Py][CFX] in $CD_3OD$.

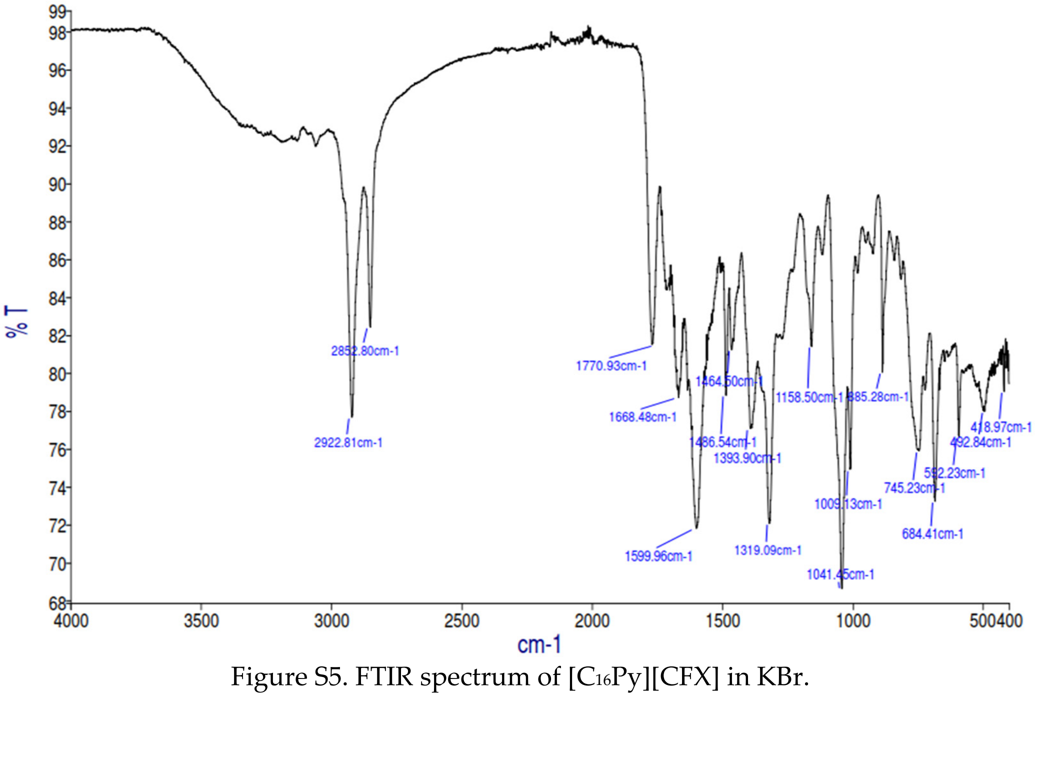


Figure S5. FTIR spectrum of [$C_{16}$Py][CFX] in KBr.

**1-Hexylpyridin-1-ium (6*S*,7*S*)-3-((carbamoyloxy)methyl)-7-((*Z*)-2-(furan-2-yl)-2-(methoxyimino)acetamido) -8-oxo-5-thia-1-azabicyclo[4.2.0]oct-2-ene-2-carboxylate, [$C_6$Py][CFX]**

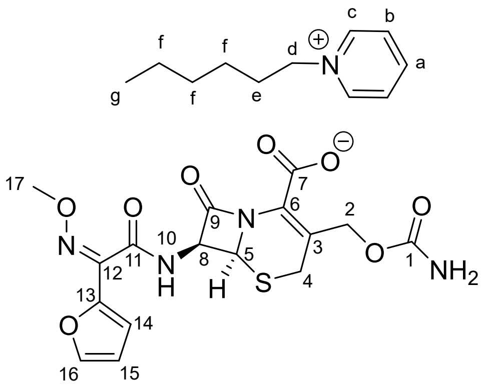

1-Hexylpyridin-1-ium hydroxide was prepared by stirring a solution of hexylpyridin-1-ium bromide (28.8 mg) with 1 mL of Amberlyst A-26(OH). Upon addition to Cefuroxime, the resulting solution was stirred for 15 minutes before evaporation and subsequent drying to provide the desired product as a dark red solid in quantitative yield (69 mg). IV (KBr) ν 3061, 2933, 2859, 1769, 1713, 1661, 1596, 1486, 1387, 1320, 1159, 1041, 1009, 885, 753, 756, 683 $cm^{-1}$. $^{1}H$ NMR (400 MHz, $CD_3OD$) δ 9.00 (d, J = 5.9 Hz, 2H, c), 8.59 (t, J = 7.8 Hz, 1H, a), 8.12 (t, J = 6.8 Hz, 2H, b), 7.64 (s, 1H, 16), 6.76 (d, J = 3.5 Hz, 1H, 14), 6.55 (br s, 1H, 15), 5.77 (d, J = 4.7 Hz, 1H, 8), 5.09 (d, J = 4.7 Hz, 1H, 5), 4.95 (d, J = 12.4 Hz, 1H, 2a), 4.79 (d, J = 12.4 Hz, 1H, 2b), 4.64 (t, J = 7.6 Hz, 2H, d), 3.97 (s, 3H, 17), 3.61 (d, J = 17.9 Hz, 1H, 4a), 3.35 (d, J = 17.9 Hz, 1H, 4b), 2.02 (q, J = 6.8 Hz, 2H, e), 1.44 – 1.29 (m, 6H, f), 0.91 (t, J = 6.6 Hz, 3H, g). $^{13}C$ NMR (101 MHz, $CD_3OD$) δ 168.9, 164.4, 164.2, 147.1, 146.9, 146.3, 146.2, 145.9, 133.7, 131.6, 129.5, 123.0, 116.9, 115.4, 114.2, 112.8, 112.8, 65.4, 63.1, 63.0, 62.3, 60.1, 58.7, 58.1, 32.4, 32.3, 29.8, 26.8, 26.7, 23.5, 14.2. Analysis calcd. for $C_{27}H_{33}N_5O_8S(H_2O)_{2.5}$: C, 51.26; H, 6.05; N, 11.07; found: C, 51.51; H, 6.03; N, 10.66.

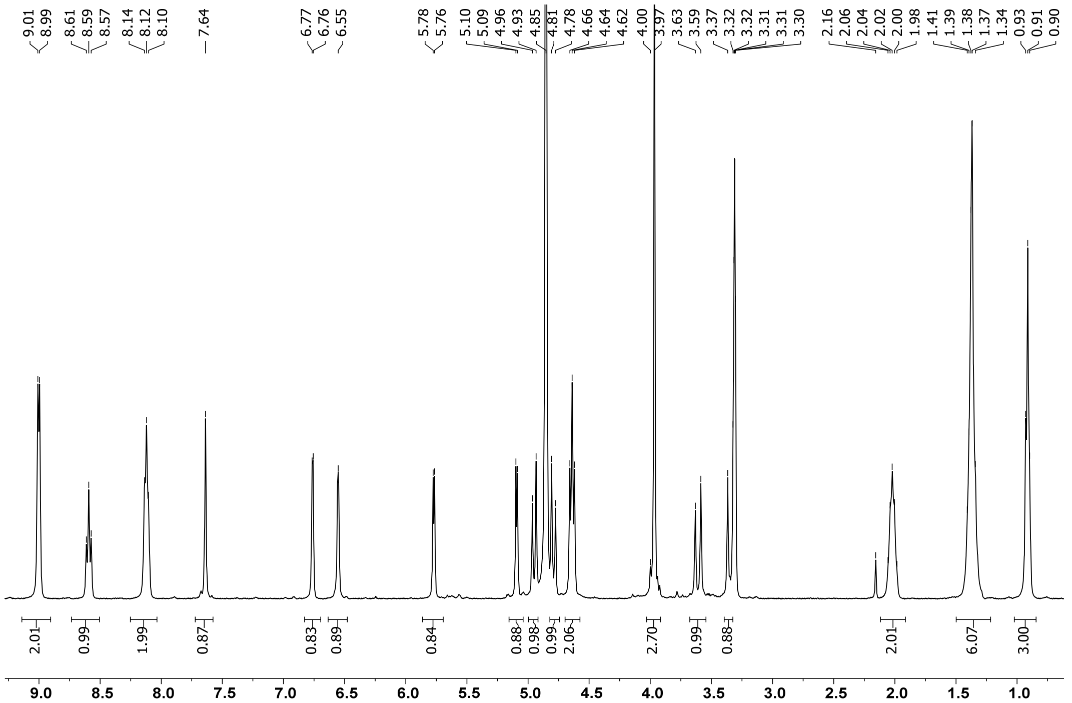

Figure S6. $^{1}H$ NMR spectrum of [$C_6$Py][CFX] in $CD_3OD$.

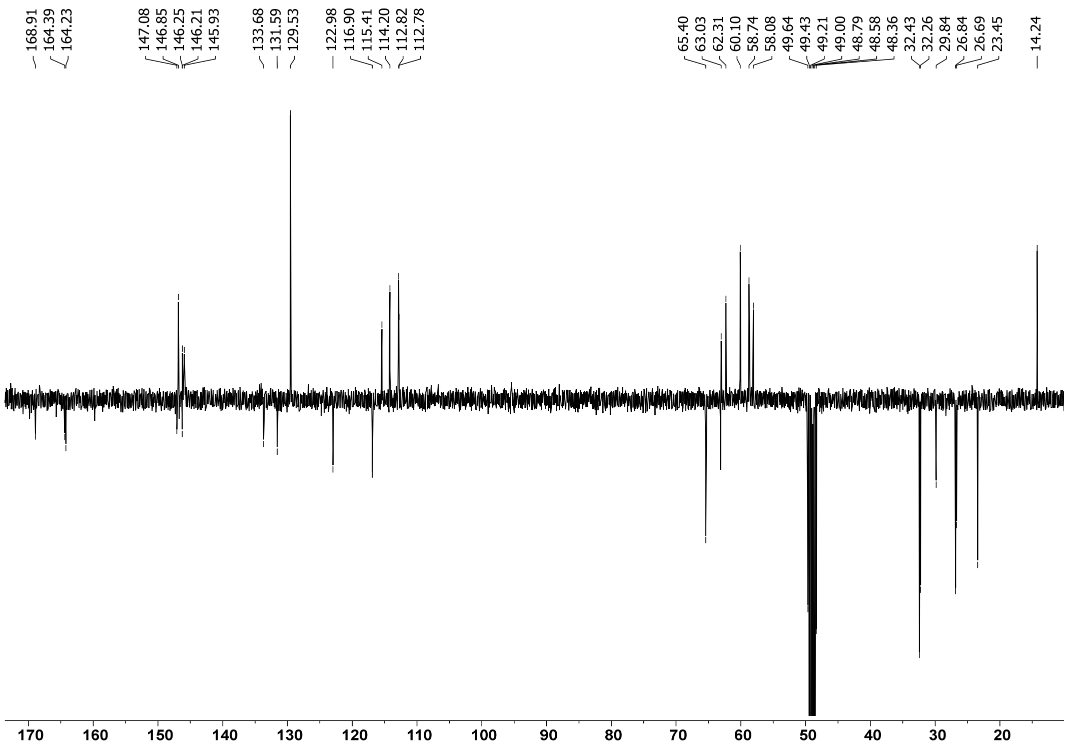


Figure S7. $^{13}$C NMR-ATR spectrum of $[C_6Py][CFX]$ in $CD_3OD$.

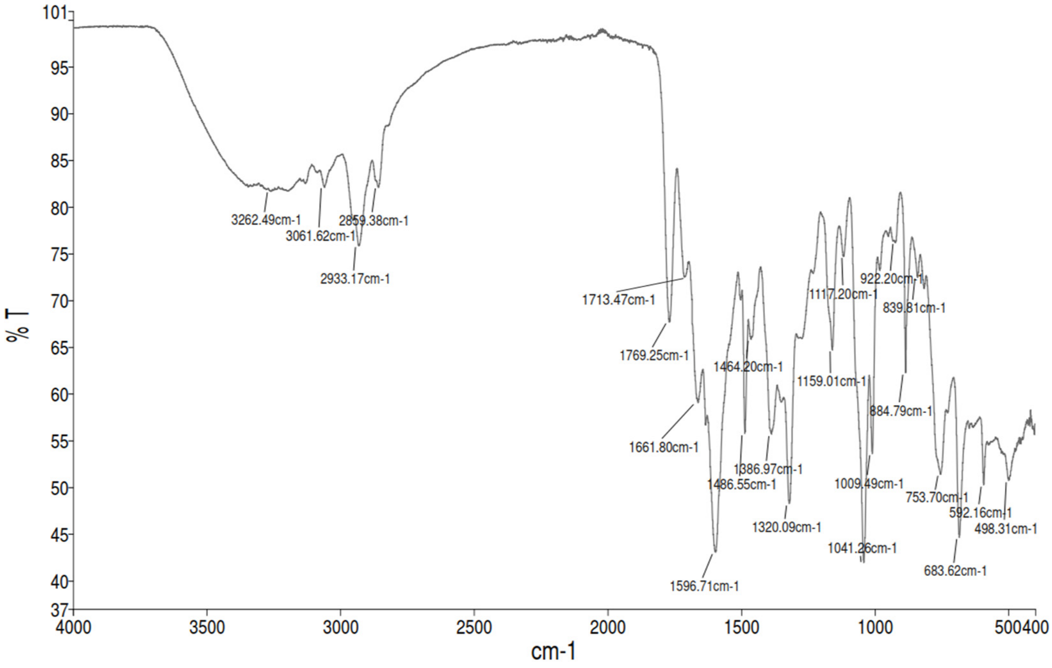


Figure S8. FTIR spectrum of $[C_6Py][CFX]$ in KBr.

**1-Decylpyridin-1-ium (6*S*,7*S*)-3-((carbamoyloxy)methyl)-7-((*Z*)-2-(furan-2-yl)-2-(methoxyimino)acetamido)-8-oxo-5-thia-1-azabicyclo[4.2.0]oct-2-ene-2-carboxylate, $[C_{10}Py][CFX]$**

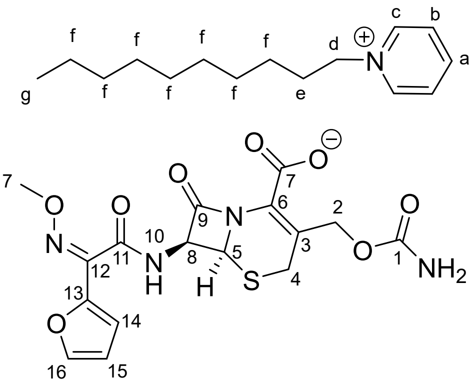


1-Decylpyridin-1-ium hydroxide was prepared by stirring a solution of decylpyridin-1-ium bromide (35.4 mg) with 1 mL of Amberlyst A-26(OH). Upon addition to Cefuroxime, the resulting solution was stirred for 15 minutes before evaporation and subsequent drying to provide the desired product as a dark red solid in quantitative yield (86 mg). IV (KBr) ν 3061, 2925, 2854, 2925, 1770, 1661, 1597, 1486, 1377, 1321, 1159, 1041, 1009, 748, 683 cm$^{-1}$. $^{1}$H NMR (400 MHz, $CD_3OD$) δ 9.00 (d, J = 5.9 Hz, 2H, c), 8.59 (t, J = 7.8 Hz, 1H, a), 8.12 (t, J = 6.8 Hz, 2H, b), 7.64 (s, 1H, 16), 6.76 (d, J = 3.5 Hz, 1H, 14), 6.55 (br s, 1H, 15), 5.77 (d, J = 4.7 Hz, 1H, 8), 5.09 (d, J = 4.7 Hz, 1H, 5), 4.95 (d, J = 12.5 Hz, 1H, 2a), 4.79 (d, J = 12.5 Hz, 1H, 2b), 4.64 (t, J = 7.6 Hz, 1H, d), 3.97 (s, 3H, 17), 3.61 (d, J = 17.8 Hz, 1H, 4a), 3.35 (d, J = 17.8 Hz, 1H, 4b), 2.02 (t, J = 7.4 Hz, 1H, e), 1.47 – 1.21 (m, 15H, f), 0.90 (t, J = 6.5 Hz, 3H, g). $^{13}$C NMR (101 MHz, $CD_3OD$) δ 168.9, 164.4, 164.2, 159.8, 147.1, 146.8, 146.3, 146.2, 145.9, 133.7, 129.5, 116.9, 114.2, 112.8, 65.4, 63.1, 60.1, 58.7, 33.0, 32.5, 30.6, 30.5, 30.4, 30.1, 27.2, 26.7, 23.7, 14.4. Analysis calcd. for $C_{31}H_{41}N_5O_8S(H_2O)_3$: C, 53.36; H, 6.79; N, 10.04; found: C, 53.51; H, 6.74; N, 10.50.

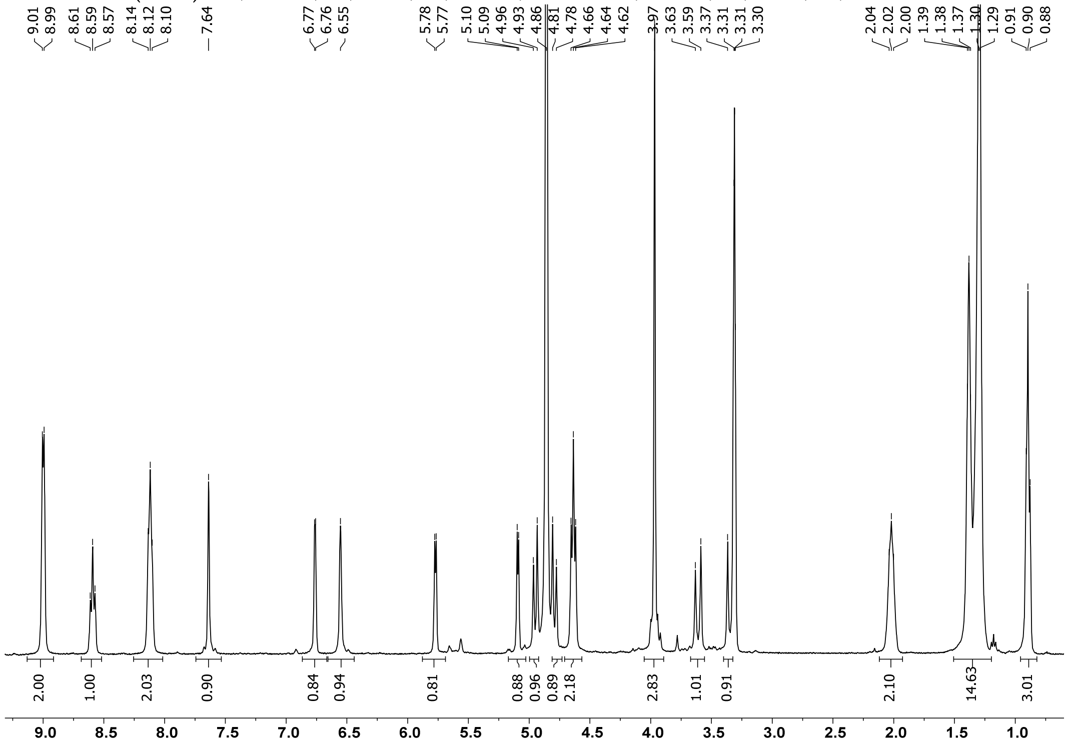

Figure S9. $^{1}$H NMR spectrum of [$C_{10}$Py][CFX] in $CD_3OD$.

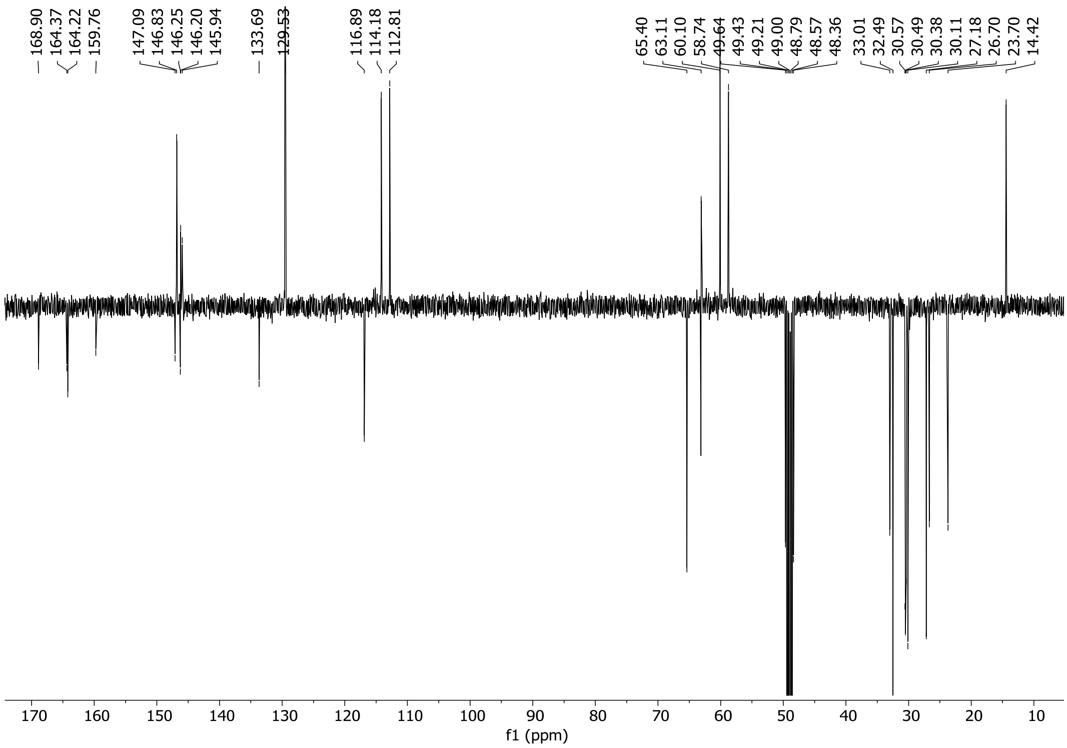


Figure S10. $^{13}C$ NMR-ATR spectrum of $[C_{10}Py][CFX]$ in $CD_3OD$.

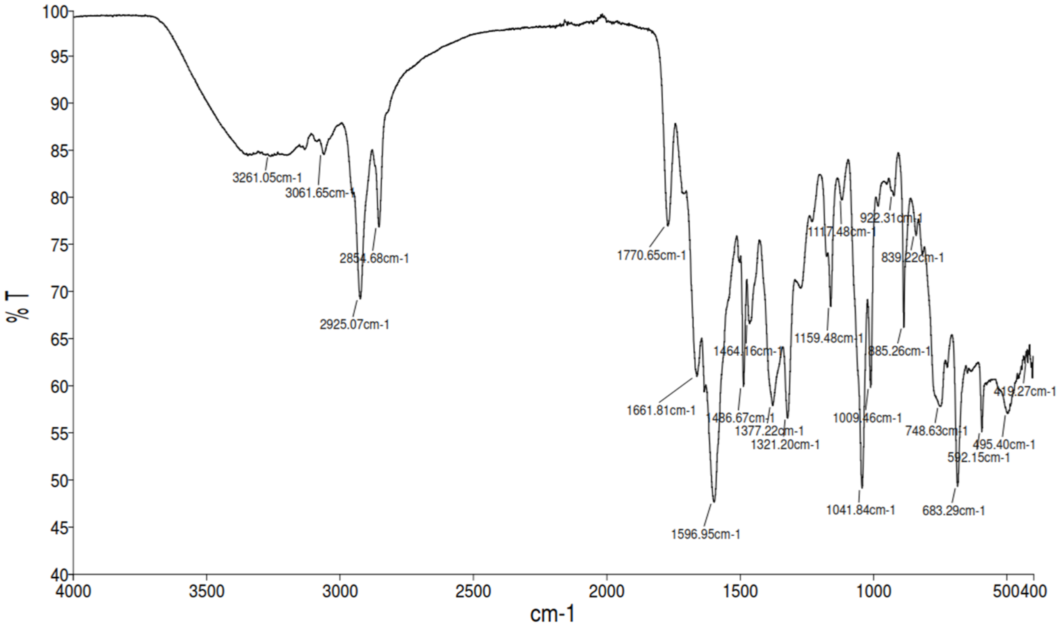


Figure S11. FTIR spectrum of $[C_{10}Py][CFX]$ in KBr.

**1-Hexadecyl-3-methylimidazolium (6*S*,7*S*)-3-((carbamoyloxy)methyl)-7-((*Z*)-2-(furan-2-yl)-2-(methoxyimino) acetamido)-8-oxo-5-thia-1-azabicyclo[4.2.0]oct-2-ene-2-carboxylate, $[C_{16}MIM][CFX]$**

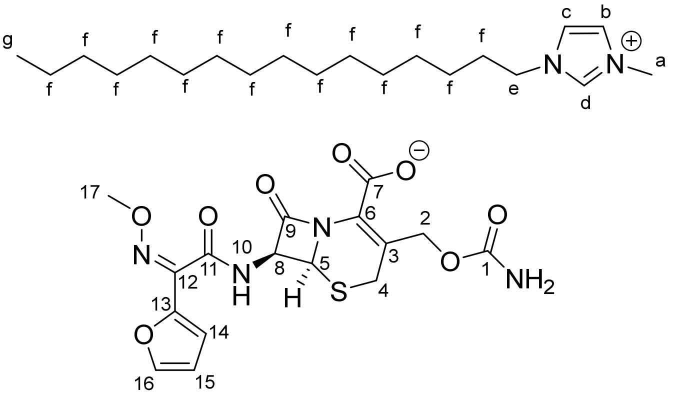

1-Hexadecyl-3-methylimidazolium hydroxide was prepared by stirring a solution of 1-hexadecyl-3-methylimidazolium bromide (45.9 mg) with 1 mL of Amberlyst A-26(OH). Upon addition to Cefuroxime, the resulting solution was stirred for 15 minutes before evaporation and subsequent drying to provide the desired product as a dark red solid in quantitative yield (86 mg). IV (KBr) ν 2922, 2852, 1770, 1698, 1600, 1486, 1393, 1319, 1158, 1041, 1009, 885, 745, 684 $cm^{-1}$. $^{1}H$ NMR (400 MHz, $CD_3OD$) δ 8.94 (s, 1H, d), 7.63 (s, 1H, 16), 7.62 (s, 1H, b), 7.56 (s, 1H, c), 6.76 (d, J = 3.4 Hz, 1H, 14), 6.55 (br s, 1H, 15), 5.77 (d, J = 4.7 Hz, 1H, 8), 5.10 (d, J = 4.7 Hz, 1H, 5), 4.96 (d, J = 12.4 Hz, 1H, 2a), 4.80 (d, J = 12.4 Hz, 1H, 2b), 4.20 (t, J = 7.4 Hz, 2H, d), 3.97 (s, 3H, 17), 3.92 (s, 3H, a), 3.61 (d, J = 17.9 Hz, 1H, 4a), 3.35 (d, J = 17.9 Hz, 1H, 4b), 1.88 (t, J = 7.2 Hz, 2H, e), 1.42 – 1.21 (m, 26H, f), 0.90 (t, J = 6.5 Hz, 3H, g). $^{13}C$ NMR (101 MHz, $CD_3OD$) δ 173.7, 170.8, 169.8, 164.9, 164.2, 147.3, 146.7, 146.3, 146.2, 146.0, 133.7, 131.6, 125.0, 123.6, 122.9, 116.9, 115.4, 114.2, 112.8, 112.8, 65.4, 63.1, 63.0, 62.3, 60.1, 58.8, 58.1, 50.8, 36.4, 33.1, 31.1, 30.8, 30.7, 30.7, 30.7, 30.6, 30.6, 30.5, 30.4, 30.1, 29.8, 27.3, 26.7, 23.7, 14.4. Analysis calcd. for $C_{36}H_{54}N_6O_8S(H_2O)_{3.5}$: C, 54.46; H, 7.74; N, 10.58; found: C, 54.35; H, 7.41; N, 10.97.

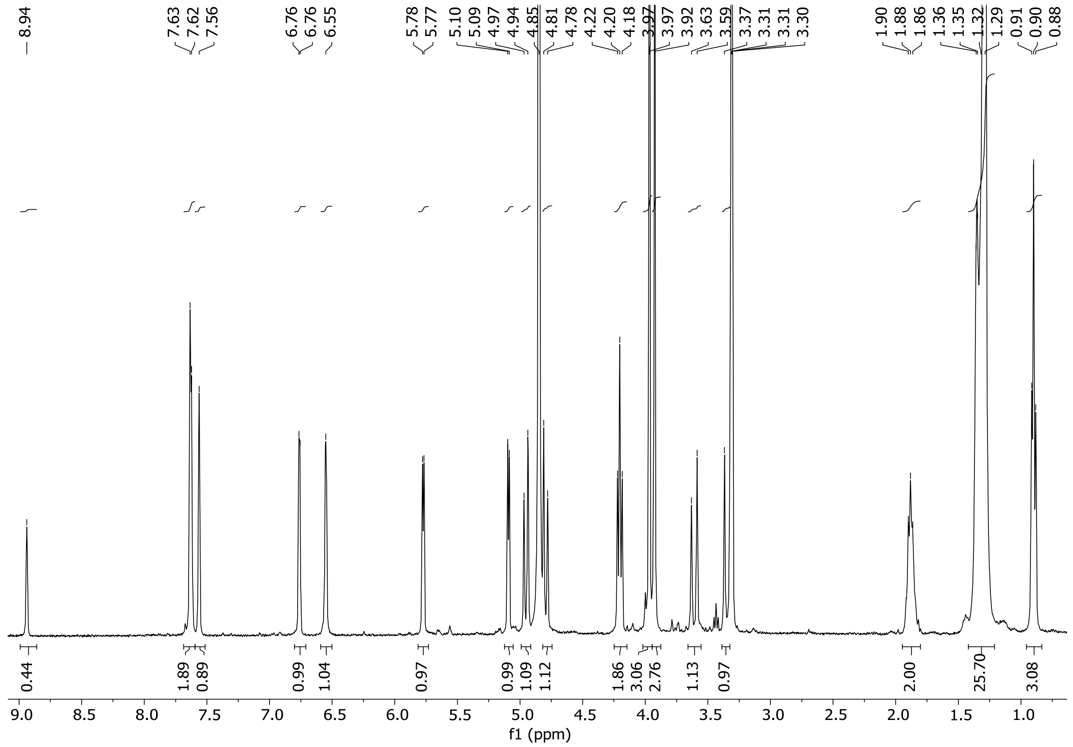


Figure S12. $^{1}H$ NMR spectrum of $[C_{16}MIM][CFX]$ in $CD_3OD$.

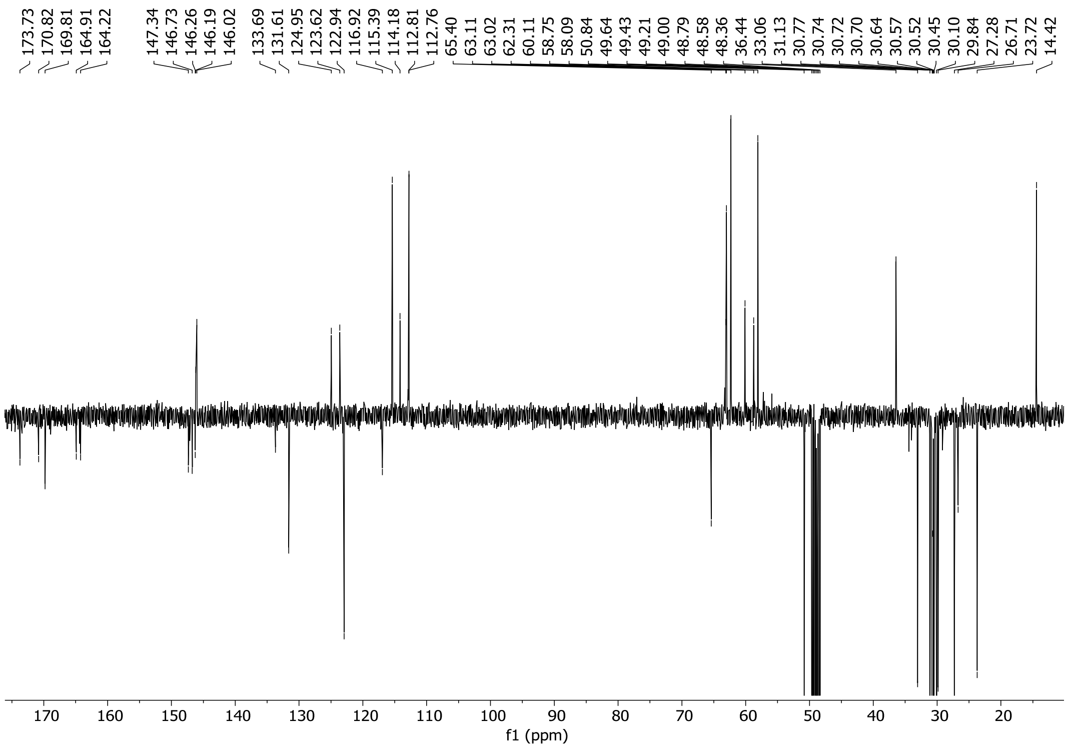


Figure S13. $^{13}C$ NMR-ATR spectrum of $[C_{16}MIM][CFX]$ in $CD_3OD$.

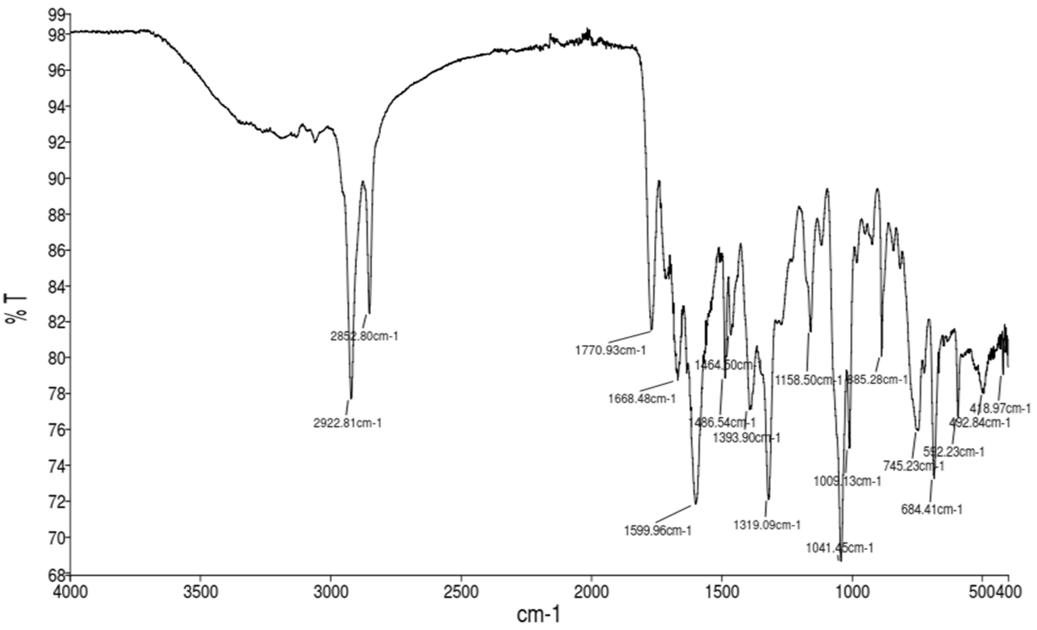


Figure S14. FTIR spectrum of $[C_{16}MIM][CFX]$ in KBr.

**1,1'-decane-1,10-diyldipyridinium bis((6*S*,7*S*)-3-((carbamoyloxy)methyl)-7-((*Z*)-2-(furan-2-yl)-2-(methoxyimino)acetamido)-8-oxo-5-thia-1-azabicyclo[4.2.0]oct-2-ene-2-carboxylate), [PyC10Py][CFX]2**

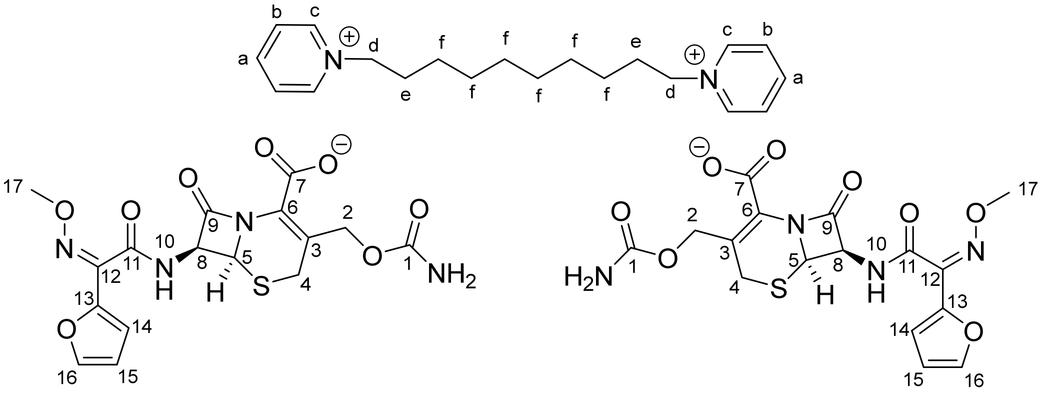

1,1'-decane-1,10-diyldipyridinium hydroxide was prepared by stirring a solution of 1,1'-decane-1,10-diyldipyridinium bromide (27.0 mg) with 1 mL of Amberlyst A-26(OH). Upon addition to Cefuroxime, the resulting solution was stirred for 15 minutes before evaporation and subsequent drying to provide the desired product as a dark red solid in quantitative yield (135 mg). IV (ATR) ν 2932, 2856, 1767, 1713, 1669, 1600, 1486, 1390, 1317, 1157, 1039, 1009, 754, 683 $cm^{-1}$. $^{1}H$ NMR (400 MHz, $CD_3OD$) δ 9.00 (d, J = 5.9 Hz, 4H, c), 8.59 (t, J = 7.8 Hz, 2H, a), 8.12 (t, J = 7.0 Hz, 4H, b), 7.64 (s, 2H, 16), 6.76 (d, J = 3.5 Hz, 2H, 14), 6.55 (br s, 2H, 15), 5.77 (d, J = 4.7 Hz, 2H, 8), 5.09 (d, J = 4.7 Hz, 2H, 5), 4.95 (d, J = 12.4 Hz, 2H, 2a), 4.79 (d, J = 12.4 Hz, 2H, 2b), 4.64 (t, J = 7.6 Hz, 4H, d), 3.97(s, 6H, 17), 3.61 (d, J = 7.3 Hz, 2H, 4a), 3.35 (d, J = 7.3 Hz, 2H, 4b), 2.07 – 1.94 (m, 4H, e), 1.44 – 1.27 (m, 12H, f). $^{13}C$ NMR (126 MHz, $CD_3OD$) δ 168.9, 164.4, 164.2, 159.8, 147.1, 146.9, 146.3, 146.2, 145.9, 133.7, 129.5, 122.9, 117.0, 115.4, 114.2, 112.8, 65.4, 63.1, 63.0, 62.3, 60.1, 58.8, 58.1, 32.5, 30.4, 30.1, 27.2, 26.7. Analysis calcd. for $C_{52}H_{60}N_{10}O_{16}S_2(H_2O)_{4.5}$: C, 50.93; H, 5.67; N, 11.42; found: C, 50.95; H, 5.58; N, 11.17.

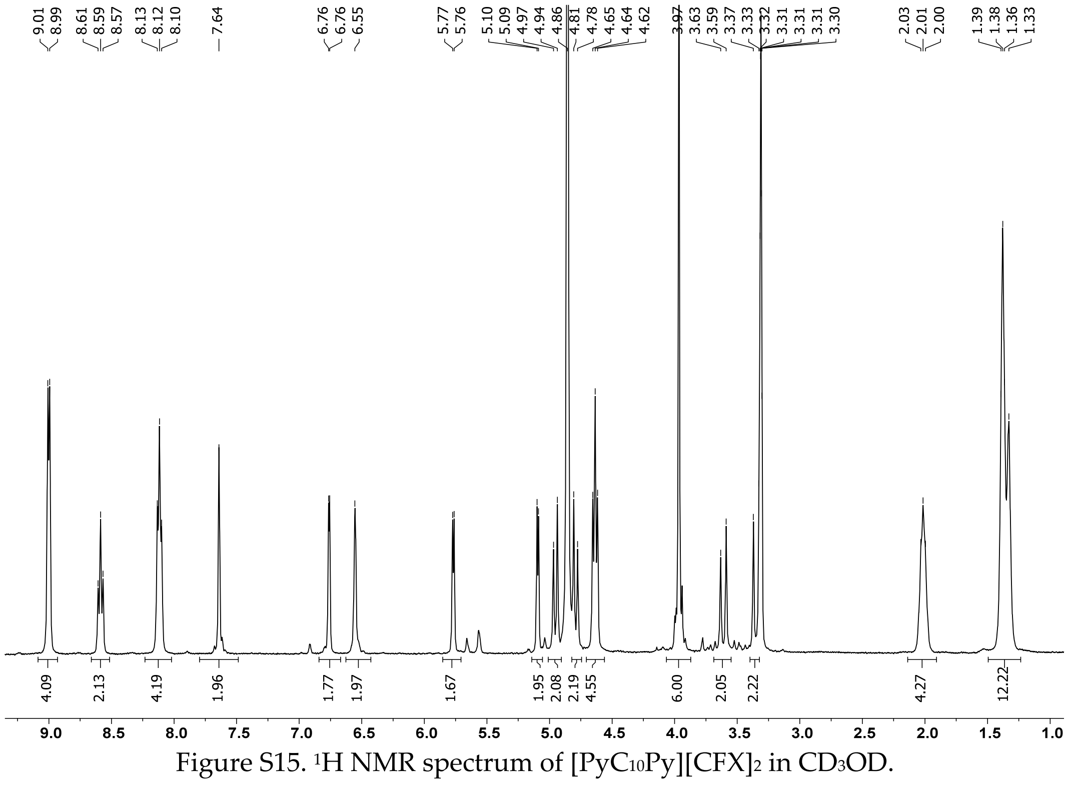

Figure S15. $^{1}H$ NMR spectrum of $[PyC_{10}Py][CFX]_2$ in $CD_3OD$.

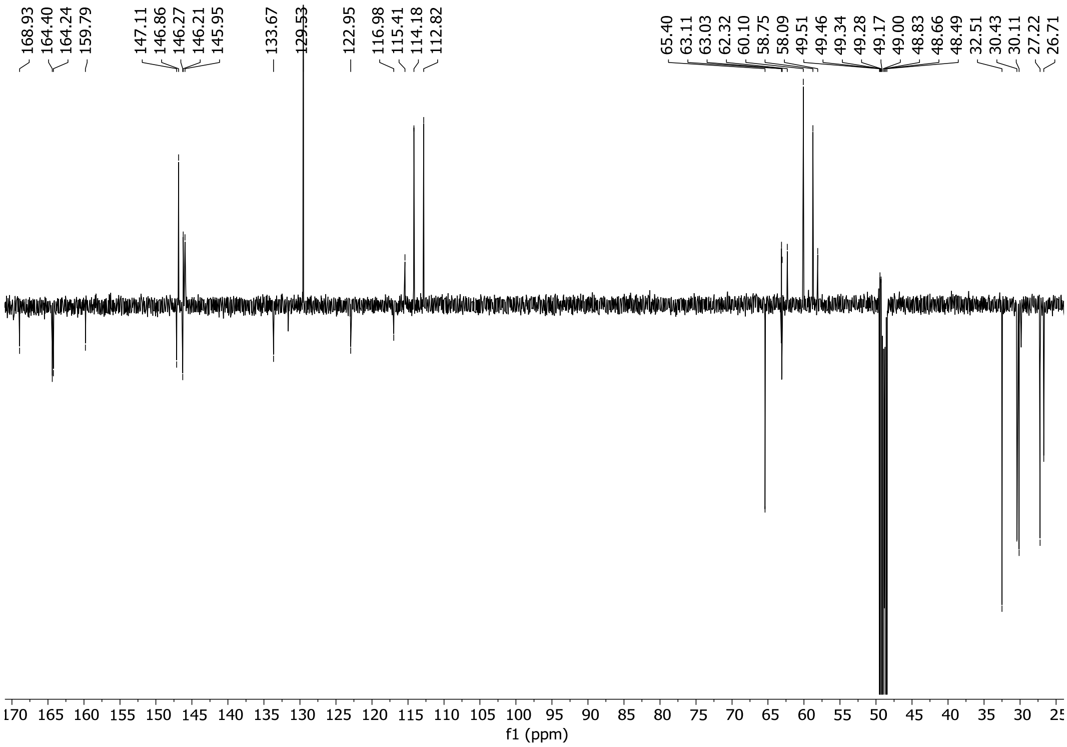


Figure S16. $^{13}C$ NMR-ATR spectrum of $[PyC_{10}Py][CFX]_2$ in $CD_3OD$.

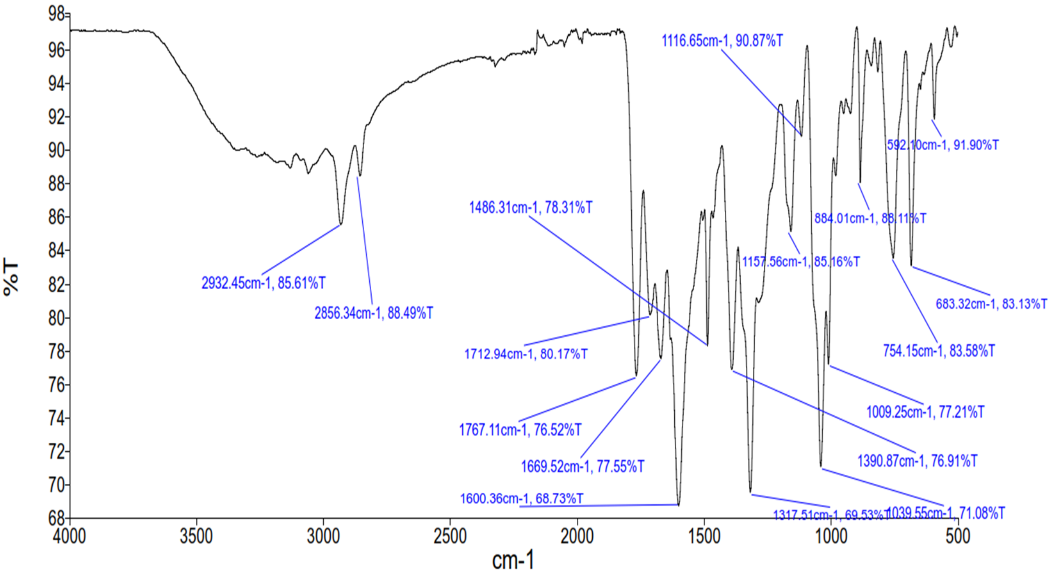


Figure S17. FTIR-ATR spectrum of $[PyC_{10}Py][CFX]_2$.

## Water solubility studies

1-5 mg of each CFX-OSIL was weighed on a 2 mL Eppendorf vial, which was then set to 37 °C. Thermostatized water was added to the Eppendorf vials 2-10 µL at a time while dissolution was observed at naked eye. Vortex and ultrasounds were periodically used to aid in solid dispersion.

## Octanol-water partition coefficient studies

1 mg of each CFX-OSIL was dissolved in 4mL of *n*-octanol saturated water in 15 mL falcon vials. From each solution, 1 mL was retrieved to serve as initial sample, while to the remainder 3 mL

solutions were added 3 mL of water saturated *n*-octanol. These heterogeneous solutions were stirred vigorously for 2 hours and then centrifuged for 10 minutes at 5000 rpm. 1 mL was retrieved from the resulting aqueous layer as the final sample. In triplicate for each sample, 200 µL were collected and diluted to 3 mL UV-Vis cells with *n*-octanol saturated water. Each sample was then analyzed in triplicate using a UV-Vis Spectrophotometer in the 400-250 nm range, and the maximum absorbance was recorded.

If precipitation at the aqueous-organic interface occurred, 0.5 mg of the CFX-OSIL were weighed instead of 1 mg, and 6 mL of water and *n*-octanol were used for the experiment. From these, 600 µl were collected to each cell.

Finally, the partition coefficients were calculated according to the following expression:

$$K_{OW} = \frac{(A_i \times df_i - A_f \times df_f)V_{\text{water}}}{A_f \times df_f \times V_{\text{octanol}}}$$
